# Tailoring Unusual Ferrimagnetism in Rare‐Earth Iron Garnets via Graphene Interlayers

**DOI:** 10.1002/advs.202506085

**Published:** 2025-09-03

**Authors:** Rui Yu, Jiefeng Cao, Fangyuan Zhu, Xiangyu Meng, Yamei Wang, Junqin Li, Yong Wang

**Affiliations:** ^1^ Shanghai Synchrotron Radiation Facility Shanghai Advanced Research Institute Chinese Academy of Sciences Shanghai 201204 P. R. China

**Keywords:** compensated ferrimagnets, graphene interlayers, magnetization compensation point, orbital moments, X‐ray absorption spectroscopy

## Abstract

Ferrimagnets (FiMs), particularly compensated FiMs, composing of coupled sublattices with antiparallel and inequivalent magnetic moments, present a unique material platform for the regulation of magnetism, which is highly desirable for the design of next‐generation spin‐based devices. Nevertheless, highly efficient methods for controlling its ferromagnetism remains significantly limited owning to the epitaxial growth required for producing high quality and fully featured films. This study, demonstrates the multiple tunability of ferrimagnetism in the rare‐earth iron garnets (REIG: thulium iron garnet) film by incorpoating the graphene interlayers. Continuous evolution of magnetic anisotropy and an unexpected/tunable magnetization compensation point (T_M_) are realized. Through soft X‐rays absorption spectroscopy analysis, the presented anisotropic behavior of orbital moments provides direct evidence for the modulation of magnetic anisotropy with large tunability. The large enhancement of the emerged T_M_ is further confirmed by the temperature‐dependent X‐ray magnetic circular dichroism signals, which reveal tunable exchange coupling for inequivalent magnetic atoms. These results establish an efficient strategy to tailor the magnetism in low dimensional REIG films through interlayer engineering and advance the study of REIG‐based spintronics.

## Graphical abstract

This study presents an efficient strategy to tailor the ferrimagnetism, such as tunable orbital moments and unexpected magnetization compensated point in low‐dimensional Tm_3_Fe_5_O_12_ films through graphene interlayer engineering. These results pave the way for spintronics based on rare‐earth iron garnets and research in orbitronics.

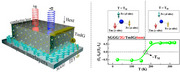

## 1 Introduction

Ferrimagnetic insulators (FiMI), which have the advantages of high Curie temperature, ultralow damping, and accessible in‐plane or perpendicular magnetic anisotropy are promising candidates for realizing low‐dissipation spin‐based devices and circuits.^[^ ^1^, ^2^, ^3^, ^4^, ^5^ ^]^ As a typical representative, rare‐earth‐based iron garnets (REIG: RE_3_Fe_5_O_12_, containing RE elements such as Thulium (Tm), Gadolinium (Gd), and Terbium (Tb)), combine the advantages of ferromagnets (FMs) and antiferromagnets (AFMs) stemming from their inherent properties, which have furnished numerous opportunities to explore exotic physical phenomena in spin community.^[^ ^6^, ^7^, ^8^ ^]^ For instance, antiparallel sublattices allow to investigate an AFMs‐like spin dynamics,^[^ ^9^ ^]^ antisymmetric exchange interaction,^[^ ^10^ ^]^ and chiral spin texture^[^ ^11^, ^12^, ^13^ ^]^ to realize fast device operation. The two inequivalent magnetic atoms in these sublattices, with distinct occupied sites and temperature‐dependent magnetization, result in highly variable and tunable magnetism, which is widely used for information storage and processing with low power consumption, high performance, and long endurance.^[^ ^6^, ^7^ ^]^ In particular, the vanishing net magnetization and angular momentum at specified temperature points are referred to one category of fully compensated REIG materials, and the corresponding points are defined as the magnetization compensation point (T_M_) and angular momentum compensation point (T_A_), respectively. The physical parameters (such as damping, dynamic frequency, domain wall velocity) in fully compensated REIG present a vanishing or diverging feature at these two‐compensation points^[^ ^14^, ^15^ ^]^ and various novel discoveries are further associated with T_M_ and T_A_, such as efficient spin‐orbit torque switching,^[^ ^16^, ^17^, ^18^ ^]^ ultrafast domain wall motion,^[^ ^19^, ^20^, ^21^ ^]^ and nano‐size skyrmions.^[^ ^22^ ^]^ Therefore, exploring the unusual spin‐related behaviors at the T_M_ and T_A_ in fully compensated FiMI materials have recently attracted intensive interests and present a unique material platform for realizing ultrafast spintronics memory and logic devices.

In previous studies, significant progress has been made in controlling the ferrimagnetic properties of fully compensated REIG materials, such as GdIG and TbIG. These advancements include tuning the compensation temperature (T_M_), saturation magnetization, and magnetic anisotropy through various method, such as epitaxial strain,^[^ ^23^ ^]^ or piezo‐strain,^[^ ^24^ ^]^ by altering the chemical composition ratio,^[^ ^25^ ^]^ element substitute.^[^ ^26^, ^27^, ^28^ ^]^ Among these approaches, the integration of fully compensated REIG films with ferroelecric (FE) substrates has shown highly efficient modualtion magnetic properties and presents a promising route for certain applicaitons. However, due to the inherently polycrystalline nature of REIG films grown on FE substrates, the modulation is largely limited to in‐plane magnetic anisotropy, which restrict their applicability.^[^ ^29^ ^]^ Therefore, achieving epitaxial growth while maintaining the fully compensated characteristice of REIG films remians a significant challenge and is still rarely reported. In addition, direct observations of the magnetization flip and the change in its magnitude for the two distinct elements at the modulated T_M_, as previously reported, still lack, which may obscure the underlying mechanism of tunable ferrimagnetism in compensated REIG films.

In this study, we report the realization of the consecutive modulation of magnetic anisotropy from perpendicular to in‐plane anisotropy and a detectable/tunable T_M_ in ultrathin TmIG films via the graphene interlayers. TmIG features three individual sublattices: Tm^3+^ ions occupying dodecahedral sites (*c*‐sites), which are ferromagnetically coupled to Fe^3+^ ions at octahedral sites (*a*‐sites), and Fe^3+^ ions at tetrahedral sites (*d*‐sites) which exhibit antiferromagnetic coupling.^[^ ^30^ ^]^ Although Néel et al. predicted the existed T_M_ between 4.2 and 20.4 K^[^ ^31^ ^]^ and Shao et al. reported the existed T_M_ ≈75 K in a specific sample, attributing to cation off‐stoichiometry,^[^ ^32^ ^]^ both bulk and thin‐film TmIG has been widely reported to lack the compensated properties based on theoretical calculations^[^ ^33^, ^34^ ^]^ and experimental studies.^[^ ^35^, ^36^, ^37^, ^38^ ^]^ Thus, to explore the possibly compensated and tunable ferrimagnetism in TmIG films, the graphene layers, which are widely used as an epitaxial release layers for heterointegration,^[^ ^39^, ^40^, ^41^ ^]^ were transferred to the target substrates (SGGG) before depositing the TmIG films. By varying the number of graphene layers (t_G_), a suitable and tunable strain was imposed on the TmIG films which was quantified via X‐ray diffraction (XRD) measurements with using the synchrotron‐ and lab‐based machines. By applying anomalous Hall effect (AHE)‐like measurements, the lineshapes of the AHE signals are evolute from rectangula to oblique, which corresponds to the evolution of magnetic anisotropy from out‐of‐plane to in‐plane. Temperature‐dependent AHE signals exhibited double‐sign reversal, and the related coercivity field exhibited divergent behavior, which further supported the emergence of T_M_ point in thin TmIG films. The observed anisotropy of orbital moments and sign reversal of X‐ray magnetic circular dichroism (XMCD) signals in TmIG films further revealed the modulated mechanism for its ferrimagnetism. Moreover, a giant and tunable ratio of the orbital moments (≈320% for 6 nm TmIG; ≈1370% for 12 nm TmIG) and the appeared T_M_ (≈400% enhancement for TmIG films with varying t_G_) are achieved. These results demonstrate that the incorporation of graphene interlayers is a highly efficient approach for the ferrimagnetism modulation of low‐dimensional REIG materials and even offers a promising avenue toward to orbitronics.

## 2 Results and Discussion

### 2.1 Structural Characterization

**Figure 1:**
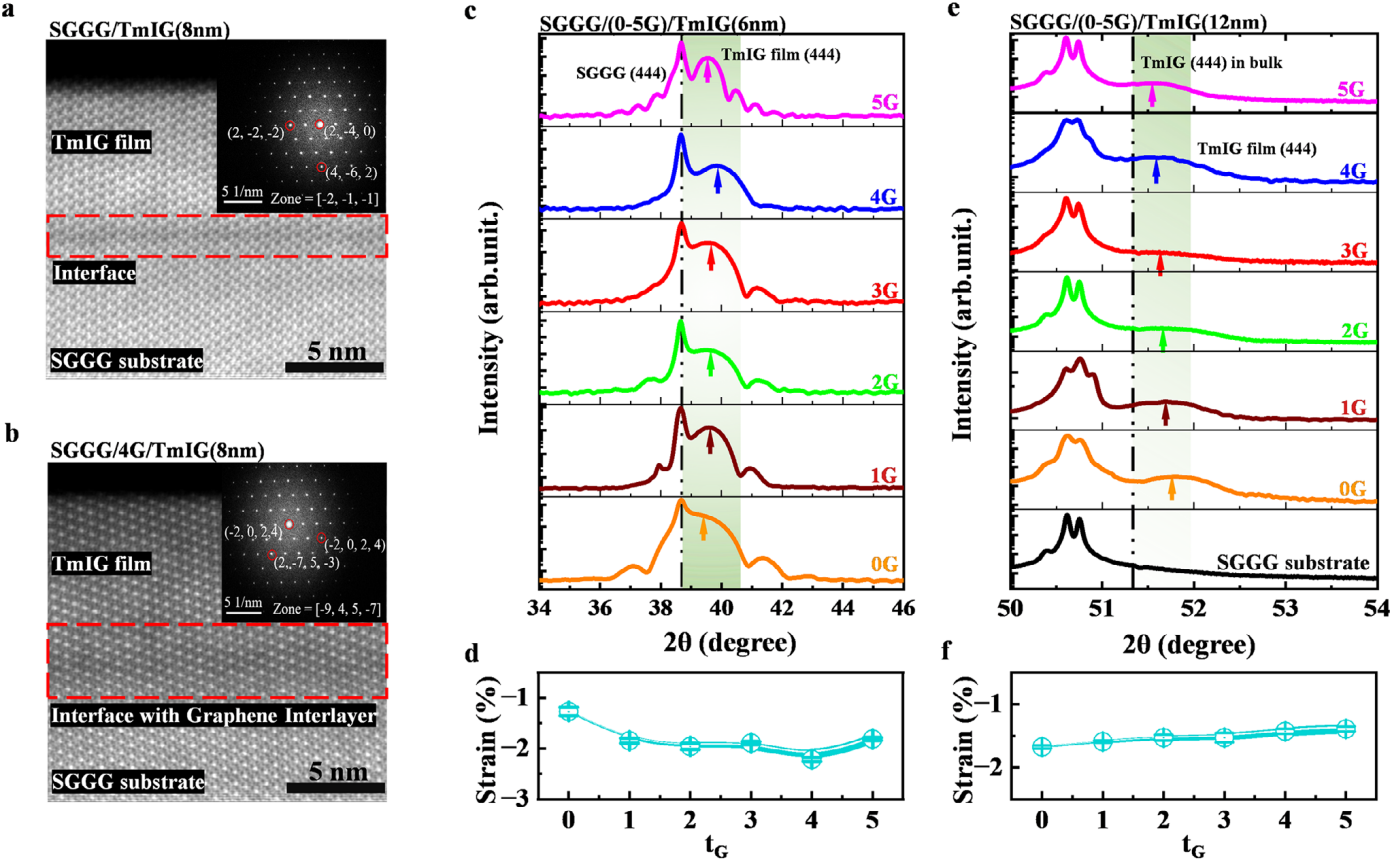
Structural characterization of the TmIG/(0–5G)/SGGG samples. a,b) The cross‐sectional TEM images of the TmIG(8 nm)/SGGG and TmIG(8 nm)/4G/SGGG interfaces, respectively. Inset shows the FFT from the image. c) XRD *θ‐2θ* scans for the TmIG(6 nm)/(0–5G)/SGGG films. These data are collected on the synchrotron‐based machines. d) The corresponding out‐of‐plane strain of TmIG(6 nm) films with 0–5 layers of graphene interlayers (t_G_). e) XRD *2θ‐ω* scans of the TmIG(12 nm)/(0–5G)/SGGG films and the data is collected on the lab‐based machines. f) The corresponding out‐of‐plane strain of TmIG(12 nm) films with 0–5 layers of graphene interlayers.

In this study, TmIG films with different thickness (TmIG(6 or 12 nm)) were deposited on SGGG substrates and 1–5 layers of graphene (1–5G) coated SGGG substrates, respectively. The transfer process and Raman spectra after the transfer of graphene are shown in Figure S1 (Supporting Information). Cross‐sectional transmission electron microscopy (TEM) is performed to verify the quality of the TmIG films, and the results are presented in **Figure** **1**a,b. A sharp interface (framed with a dashed line) and visible diffraction pattern (inset) were observed for SGGG/TmIG(8 nm) and SGGG/4G/TmIG(8 nm), respectively. This indicates that TmIG films with excellent crystallinity were obtained for both TmIG films with or without graphene interlayers. To further investigate the strain induced by graphene interlayers, the crystal structures of the TmIG films were characterized by using XRD. Specifically, for the TmIG(6 nm)/(0–5G)/SGGG films, XRD measurements were carried out using synchrotron‐based radiation source and the Figure 1c presents the detected spectra. For each spectrum, the presence of Laue oscillations further confirmed the high crystallinity of the deposited TmIG films, which was consistent with the TEM results mentioned above.^[^ ^42^, ^43^ ^]^ The main peak was for the (444) peak of the SGGG substrate as labeled. The short arrows indicated the actual positions of the (444) peaks of the TmIG films with 0–5G of graphene interlayers.^[^ ^21^ ^]^ And a comparison of these related peaks shown a non‐monotonic behavior with varying layers of graphene. This observation indicated that the graphene interlayers generated a t_G_‐dependent strain effect on the deposited TmIG(6 nm) films, and the accompanying strain was released with increasing t_G_. Moreover, the induced strain (ε) in the TmIG films due to the lattice mismatch can be estimated based on the XRD data^[^ ^44^ ^]^ (Figure S2, Supporting Information for more details). In Figure 1d, the t_G_‐dependent strain results show with a negative value, confirming the compressive nature of the strain in the TmIG films due to the graphene interlayers. Similarly, for a relatively thick TmIG film, the TmIG(12 nm)/(0–5G)/SGGG samples, the peak positions of the TmIG films presented a consecutive shift toward the (444) peaks of the SGGG substrate as shown in Figure 1e. The t_G_‐dependent strain showed gradually decreasing trend, as shown in Figure 1f. The strain value was less than in TmIG(6 nm), which intuitively reflects a weaker induced strain in the thick TmIG(12 nm) film.

### 2.2 Electrical Transport Measurements

**Figure 2:**
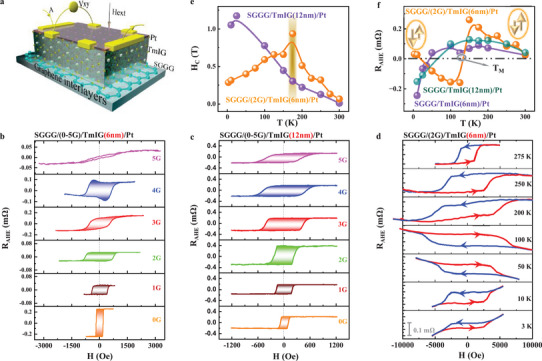
Anomalous Hall effect (AHE) measurements of the Pt/TmIG/(0–5G)/SGGG samples. a) Schematic illustrations of AHE measurement of Pt/TmIG/SGGG with graphene interlayers. b,c) R_AHE_ versus H curves of the Pt/TmIG(6 nm)/(0–5G)/SGGG and Pt/TmIG(12 nm)/(0–5G)/SGGG measured at room temperature, respectively. d) Temperature dependent R_AHE_ as a function of H for the Pt/TmIG(6 nm)/(2G)/SGGG sample. e) Temperature dependence of coercivity field (H_C_) for the Pt/TmIG(6 nm)/(2G)/SGGG (orange) and Pt/TmIG(12 nm)/SGGG (violet) samples, respectively. f) Temperature dependent R_AHE_ values of the Pt/TmIG(6 nm)/(2G)/SGGG (orange), Pt/TmIG(6 nm)/SGGG (violet) and Pt/TmIG(12 nm)/SGGG (dark cyan) samples, respectively.

Next, we performed magneto‐transport measurements to unveil the influence of the induced strain on the platinum (Pt)(6 nm)/TmIG(6 or 12 nm)/(0–5G)/SGGG samples. Notably, for ultrathin TmIG films, electrical measurement is a better fingerprint for reflecting the hysteresis behavior than the magnetic hysteresis loop measured by a vibrating sample magnetometer owing to the largely paramagnetic background from the SGGG substrate or magneto‐optic Kerr owing its transparent properties.^[^ ^32^ ^]^ **Figure** **2**a shows a schematic illustration of AHE measurement of Pt/TmIG/SGGG with graphene interlayers. For the samples Pt(6 nm)/TmIG(6 nm)/(0–5G)/SGGG, the AHE resistance R_AHE_ after subtracting the linear background by sweeping the out‐of‐plane magnetic field at room temperature (RT) conditions is presented in Figure 2b. The lineshapes of R_AHE_ are evolved from rectangular to oblique. Generally, the square represents the owned perpendicular magnetic anisotropy (PMA) in the Pt(6 nm)/TmIG(6 nm)/(0–2G)/SGGG samples. The gradually oblique lineshapes reflect the magnetic anisotropy of the TmIG films toward in‐plane anisotropy in Pt(6 nm)/TmIG(6 nm)/(3–5G)/SGGG. Specifically, a visible topological Hall‐like signal occurred at RT in Pt(6 nm)/TmIG(6 nm)/(4G)/SGGG which is possibly associated with the competing results of various energies under the strain induced by the graphene interlayers.^[^ ^13^ ^]^ Therefore, the apparent strain effect on the TmIG(6 nm) films resulted in a phase transition of the magnetic anisotropy from perpendicular to in‐plane. In contrast, for the thick TmIG(12 nm) films, the lineshapes of R_AHE_ remain rectangular with a little oblique, as shown in Figure 2c, which is reasonable with a weak strain change as presented in Figure 1f. This indicates the PMA properties are alive for Pt(6 nm)/TmIG(12 nm)/(0–5G)/SGGG samples. To further investigate the change in magnetic anisotropy, we extracted the effective PMA filed (H_K_) by performing AHE measurements with a magnetic field applied along the hard axis^[^ ^45^ ^]^ (more details are provided in Figure S3, Supporting Information). The ratios of H_K_ (1–5G) to H_K_ (0G) and R_AHE_ (1–5G) to R_AHE_ (0G) as a function of t_G_ are presented in Figure S3d (Supporting Information). A peak behavior occurred, which suggests the graphene interlayers enhanced H_K_ (R_AHE_) at a specific t_G_.

In principle, the lineshape and amplitude of the R_AHE_ is used to reflect the modulation of magnetic anisotropy induced by graphene interlayers as discussed above, whereas the sign‐reversal of the R_AHE_ has been widely considered as a benchmark to clarify the existing competition between magnetic proximity and spin Hall effect, or to reflect the moment direction at a specified temperature point in magnetic insulator/heavy metal bilayers.^[^ ^13^, ^38^ ^]^ Temperature‐dependent AHE measurements were performed on the Pt/TmIG(6 nm)/(2G)/SGGG and Pt/TmIG(6 nm)/SGGG samples. Figure 2d exhibits the typical temperature dependence of the AHE resistance with visible hysteresis behavior for all temperatures, and its sign experiences a double‐sign reversal, which occurs at ≈50 K and 150 K for the Pt/TmIG(6 nm)/(2G)/SGGG sample. The corresponding AHE measurements up to 2T are presented in Figure S4 (Supporting Information). In contrast, the temperature‐dependent R_AHE_ for the Pt/TmIG(6 nm or 12 nm)/SGGG sample shows a sign reversal at ≈50 K, which exhibits a distinguished feature with respect to the Pt/TmIG(6 nm)/(2G)/SGGG sample, as presented in Figure 2f. Generally, the sign change of the R_AHE_ at ≈50 K is attributed to the competition between magnetic proximity and spin Hall effect which had been studied intensively in REIG/Pt bilayers, such as YIG/Pt and TbIG/Pt.^[^ ^36^ ^]^ Another sign change in the R_AHE_ at ≈150 K probably stemmed from the induced T_M_ point in Pt/TmIG(6 nm)/(2G)/SGGG via graphene interlayers. In addition, as a signature feature of fully compensated REIG films, the observed temperature‐dependent coercivity field (H_C_) exhibited a divergent behavior at ≈150 K, as presented in Figure 2e, which further supports the emerging compensated property of Pt/TmIG(6 nm)/(2G)/SGGG.^[^ ^23^, ^25^ ^]^ And the H_C_ increases with decreasing temperature in Pt/TmIG(12 nm)/SGGG which is a typical phenomenon for FiMs materials. Thus, tunable magnetic anisotropy and unexpected T_M_ point were achieved by incorporating the graphene interlayers in thin TmIG films.

### 2.3 Angle‐Dependent XAS and XMCD Measurements

**Figure 3:**
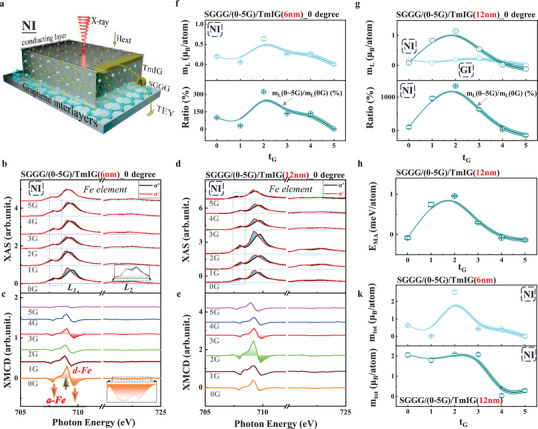
X‐ray absorption spectra (XAS) measurements of the TmIG(6 nm or 12 nm)/(0–5G)/SGGG samples. a) Schematic illustrations of XAS measurements of TmIG/SGGG with graphene interlayers under normal incidence (NI). b,d) Experimental XAS spectra of the TmIG(6 nm or 12 nm)/(0–5G)/SGGG samples at the Fe L_3,2_ edges under NI, respectively. The black and red lines show the positive helicity (+𝝈) and the negative helicity (‐𝝈), respectively. c,e) The corresponding X‐ray magnetic circular dichroism (XMCD) signals for the TmIG(6 nm or 12 nm)/(0–5G)/SGGG samples, respectively. f) t_G_‐dependent orbit moments (top) and the ratios of m_L_ (0–5G) to m_L_ (0G) (bottom) for TmIG(6 nm)/(0–5G)/SGGG samples, respectively. g) t_G_‐dependent orbit moments obtained at NI and GI geometry (top) and the ratio of m_L_ (0–5G) to m_L_ (0G) (bottom) for TmIG(6 nm)/(0–5G)/SGGG samples, respectively. h) t_G_‐dependent E_MA_ for TmIG(12 nm)/(0–5G)/SGGG samples. k) t_G_‐dependent total moments (m_tot_) for TmIG(6 nm or 12 nm)/(0–5G)/SGGG samples, respectively. All measurements are performed at room temperature.

To further investigate the graphene interlayers‐driven modulation of magnetic anisotropy, element‐specific soft X‐ray absorption spectroscopy (XAS) and XMCD measurements were carried out for the TmIG(6 or 12 nm)/(0–5G)/SGGG samples. In **Figure** **3**a, it presents a schematic of the experimental setup for the XAS measurements with total electron yield (TEY) mode at normal incidence (NI). The XAS spectra were taken at Fe L_3,2_‐edges, which gives a dominated contribution to the magnetism of the TmIG films at RT. Opposite circularly polarized X‐rays (+σ and ‐σ), with their helicity aligned to the applied magnetic field, are incident on the samples, as shown in Figure 3a. Figure 3b,d shows typical XAS signals for TmIG(6 or 12 nm)/(0–5G)/SGGG samples, respectively. A visibly distinct XAS spectra was observed with respect to the light helicity. The XAS spectra with +σ are in the edge shoulder at the L_3_ edges, whereas the XAS spectra with ‐σ are in the edge shoulder at the L_2_ edges (Figure 3b inset), which is attributed to the spin‐orbit coupling of the 2p core level and is consistent with previous reports on REIG films. Specifically, the comparison of the XAS spectra, for instance, in the dashed regions of Figure 3b, exhibit a diminishing difference of the lineshaps for +σ and ‐σ as t_G_ increases. And a similar but weak trend was observed in the TmIG(12 nm)/(0–5G)/SGGG samples, as shown in Figure 3d owning to a weak strain effect as mentioned in Figure 1f. These observations indicate that the local environments of Fe^3+^ ions located at the *a‐* and *d‐*sites are qualitatively influenced by the graphene interlayers. Furthermore, the corresponding XMCD spectra ((‐σ)‐(+σ)) were derived to reflect site‐specific information on the magnetic properties, as presented in Figure 3c,e for TmIG(6 or 12 nm)/(0–5G)/SGGG samples, respectively. The XMCD spectrum exhibits two negative peaks caused by Fe^3+^ ions at *a*‐sites and one positive peak caused by Fe^3+^ ions at *d*‐sites (as labeled in Figure 3c) which confirms the expected magnetic sublattices and antiferromagnetic coupling of Fe^3+^ ions at the *a*‐ and *d*‐sites,^[^ ^46^ ^]^ and the exchange interactions between Fe^3+^ and O^2−^ ions are similarly obtained via XAS/XMCD signals as reported.^[^ ^47^ ^]^ The opposite peaks at the L_3_ and L_2_ edges indicate its intrinsic magnetism in the TmIG films. The intensity of the XMCD signals, especially for the peak at *d*‐sites, presents a strong t_G_‐dependence, which implies a significant modulation in the magnetic moments and further generates an apparent influence on the magnetic properties of these TmIG films with graphene interlayers.

Therefore, to identify its presence, the atomic magnetic moments, including the orbital (m_L_) and spin (m_S_) magnetic moments, were obtained using the XMCD sum rules analysis, as described in Figure 3f (top panel)^[^ ^48^ ^]^ (more details are provided in Figure S5, Supporting Information). And the magnetism contribution from Fe elements a‐ and d‐sites are presented using atomic multiplet calculatioons (more detailes are presented in Figure S6, Supporting Information). In Figure 3f, the orbit moments show a highly anisotropic behavior (top panel) with respect to t_G_ in NI geometry, and its value presents a giant enhancement of up to 320% (bottom panel) with respect to t_G_ = 0G for TmIG(6 nm)/(0–5G)/SGGG samples. The orbital moments follow a similar trend in the TmIG(12 nm)/(0–5G)/SGGG samples, as presented in Figure 3g (top panel) in NI geometry, and its value presents a higher enhancement of up to 1370% (bottom panel) owing to a smaller orbital moment in a relatively thicker TmIG(12 nm)/(0G)/SGGG sample. In contrast, a small variation in the orbital moments with respect to t_G_ are observed in the TmIG(12 nm)/(0–5G)/SGGG samples in grazing incidences (GI) geometry as described in Figure 3g (bottom panel) which indicates a dominant contribution from the perpendicular orbital moment in the anisotropic behavior (more results for the GI geometry can be found in Figure S7, Supporting Information). The resulting anisotropy of the orbital moment is further related to the element‐specific magnetic anisotropy energy (*E_MA_*) based on the perturbation theory model,^[^ ^46^ ^]^ where the *E_MA_* describes the magnetization tendency to align along specific directions and is directly on the lineshape of R_AHE_ as obtained in Figure 2b,c. And the *E_MA_* is estimated by using the formula *E_MA_* ≈(ξ_Fe_/4µ_B_)(m_L_(NI)‐ m_L_(GI)), where ξ_Fe_ is the spin‐orbit coupling constant for Fe and has a value close to 50 meV; µ_B_ is the Bohr magneton;^[^ ^49^, ^50^ ^]^ m_L_(NI) and m_L_(GI) are the corresponding orbital moments at NI and GI geometry, respectively. Obviously, the *E_MA_* follows a concurrent trend with the behavior of orbital moment as described in Figure 3h (similar trend is otained for strain‐depenndent *E_MA_* as presented in Figure S8, Supporting Information), which provides an intuitive cause for the modulation of magnetic anisotropy reflected in the lineshape of the R_AHE_ signal via graphene interlayers. In addition, t_G_‐dependent total moments, for TmIG(6 nm)/(0–5G)/SGGG and TmIG(12 nm)/(0–5G)/SGGG (Figure 3k) further suggest a corresponding change in the R_AHE_ amplitude as presented in Figure 2b,c. Thus, the presented highly anisotropic orbital moment provides a direct explanation for the effective modulation of the magnetic anisotropy in TmIG thin films via the graphene interlayers.^[^ ^49^ ^]^ Moreover, to further investigate the graphene interlayers‐driven modualtion of magnetic properties, the strain‐dependent total magnetic anisotropy energy (*MAE*) is acquired via the ab initio calcualtions. The corresponding results are presented in the Figure S9 (Supporting Information). Additionally, the potential contribution to *MAE* from interfacial orbital‐hybrization^[^ ^51^, ^52^ ^]^ between graphene interlayer and TmIG is not included in the current calcuation and should be considered in future studies for a better understanding the modulatoin mechnism through graphene interlayer.

### 2.4 Temperature‐Dependent XAS and XMCD Measurements

**Figure 4:**
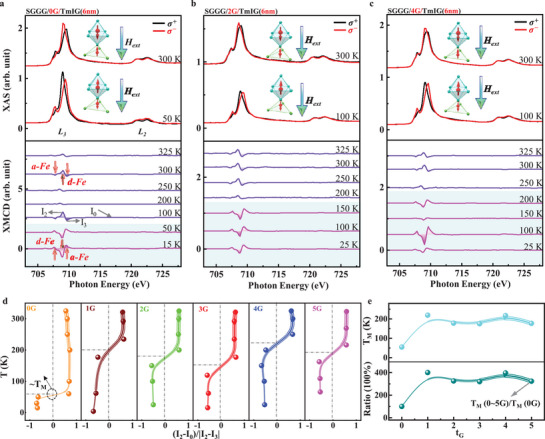
Temperature‐dependent XAS and XMCD signals in the TmIG(6 nm)/(0–5G)/SGGG samples. a‐c) XAS (top) and the corresponding XMCD signals (bottom) for these films TmIG(6 nm)/0G, TmIG(6 nm)/(2G) and TmIG(6 nm)/(4G), respectively, on SGGG substrates. Data are offset for clarity. d) Temperature‐dependent XMCD intensity ratio for 0 to 5 layers of graphene interlayers. e) The emerged T_M_ (top) and the ratio of T_M_(0–5G) to T_M_(0G) (bottom) as a function of t_G_ for TmIG(6 nm)/(0–5G)/SGGG samples, respectively. All signals are taken at NI geometry.

Furthermore, to verify the emerging T_M_ point, temperature‐dependent XAS and XMCD measurements were conducted at the Fe‐L_3,2_ edges of the TmIG(6 nm)/(0–5G)/SGGG samples. **Figure** **4**a (top panel), presents the typical XAS signals obtained with the opposite circularly polarized X‐rays ((+σ (black line) and ‐σ (red line)) for TmIG(6 nm)/(0G)/SGGG sample. At T = 300K, the XAS spectrum with ‐σ appears at the edge shoulder of the L_3_ main peak, whereas the spectrum with +σ are observed at the edge shoulder of the L_2_ main peak. In contrast, at T = 50K, the edge shoulder of the L_3_ main peak is reflected in the +σ spectra, whereas the L_2_ main peak emerges in the ‐σ spectrum. The temperature‐dependent XMCD spectra of the TmIG(6 nm)/(0G)/SGGG sample are presented in the bottom panel of Figure 4a. Flipping of the orientation occurred in the XMCD spectra within the temperature range of 50–100 K. This reversal in the XMCD spectral features reveals the magnetic moments of the Fe element occuping the *a*‐ and *d*‐sites, which undergo temperature‐driven reorientation, as illustrated in Figure 4a (top panel). Similar behavior was observed for various critical temperatures in the TmIG(6 nm)/(2G and 4G)/SGGG samples, as shown in Figure 4b,c, as well as in the TmIG(6 nm)/(1G, 3G and 5G)/SGGG samples, as detailed in Figure S10 (Supporting Information). In contrast, for thicker TmIG (12 nm) films, a reversal in the XMCD spectra occurred in the TmIG(12 nm)/(4G and 5G)/SGGG samples, as detailed in Figure S11 (Supporting Information). To highlight the reversal effect occurring at a specific temperature, the temperature dependence of the XMCD intensity ratio ((I_2_‐I_0_)/|I_2_‐I_3_|) is shown in Figure 4d for the TmIG(6 nm)/(0–5G)/SGGG samples, where the values of I_0_, I_2_, I_3_ are taken from the XMCD spectra, as labeled in Figure 4a (bottom panel). A zero‐crossing point was obtained and the corresponding temperature was referred to as the emerging T_M_ point. Additionally, for the Tm, temperature‐dependent XAS and XMCD spectra of the TmIG(6 nm)/(2G)/SGGG sample were obtained (see Figure S12, Supporting Information for details). A similar orientation flipping in the XMCD spectra were observed as the temperature varied. In comparison, the orientation of the XMCD spectra for Tm elements at the M_5_‐edge is consistently opposite to that of Fe^3^⁺ ions on *d*‐sites, whereas it aligns with Fe^3^⁺ ions on *a*‐sites over a wide temperature range. The coupling is immune to external magnetic fields around the T_M_ point^[^ ^14^ ^]^ (see Figure S13, Supporting Information for details). These results are consistent with the characteristics of fully compensated REIG materials,^[^ ^6^, ^7^, ^14^ ^]^ indicating the emergence of T_M_ points in the TmIG(6 nm)/(0–5G)/SGGG samples. Moreover, as anticipated, the total magnetization shows a zero‐crossing point as a function of temperature^[^ ^14^, ^15^ ^]^ (see Figure S14, Supporting Information for details), which further confirms the presence of T_M_ points in the TmIG(6 nm)/(0–5G)/SGGG samples. In addition, Figure 4e (top panel) illustrates the unexpected T_M_ as a function of t_G_ and a significant enhancement of its value by up to 400% with respect to the TmIG(6 nm)/(0G)/SGGG sample was achieved, as presented in Figure 4e (bottom panel). Thus, the emergence of the T_M_ point was verified through temperature‐dependent XAS and XMCD measurements in low‐dimensional TmIG films, and a giant modulation was realized via the graphene interlayers.

## 3 Conclusion

We have successfully fabricated high‐quality TmIG thin films on single‐crystal SGGG substrates coated with 0–5 monolayers of graphene. Owing to the induced strain at the interface between TmIG and SGGG/(0–5G), considerable modulation of the magnetic anisotropy and an unexpected T_M_ point were achieved. In combination with temperature‐dependent XAS and XMCD measurements, highly anisotropic orbital moments with respect to t_G_ present direct evidence for the tunability of magnetic anisotropy, whereas the compensated features are verified by the flipping of the orientation in the XMCD spectra and the vanishing magnetic moments at a specific temperature. Accordingly, large enhancements in the orbital moments and T_M_ were realized. These achievements demonstrate the importance of investigating ferrimagnetism in low dimensional REIG materials to advance the study of REIG‐based spintronics. In addition, while graphene interlayers can effectively suppress atomic interdiffusion during TmIG films preparation, enable remote epitaxial growth, and simultaneously introduce suitable strain to modulate the magentic properties of TmIG. They also offer the potential for fabricating freestanding, single‐crystalline TmIG films with PMA in future studies. However, the limited tunability and nonmonotomic control currently constrain its practical utility. Thus, a further investigation is needed for the next.

## 4 Experimental Section

### Sample Fabrication and Characterization

To transfer the graphene layer to the target substrate, the graphene grown on copper foil was used and coated with PMMA. The copper foil was then immersed in ammonium persulfate solution and etched. The graphene with PMMA was then fully transferred onto a (111)‐oriented SGGG (0.5 mm) substrate via immersing in acetone. The TmIG film was deposited onto a SGGG substrate coated with various layers of graphene using radio frequency (RF) magnetron sputtering from a TmIG target at RT. During the depositoin process, the base pressure was better than 5×10^−8^ Torr, the working Argon (Ar) pressure was stable at 3 mTorr and the RF power was set to 65 W. The deposition rate of the TmIG was 0.88 nm min^−1^. After deposition, the samples were annealed in air atmosphere at 1100 K for 2 h to recrystallize the TmIG films. A Hall‐bar like Platinum (Pt) stripe was deposited on the TmIG film by DC‐magnetron sputtering with a working Ar pressure of 3 mTorr, and a DC power of 20 W at RT. The deposition rate of the Pt film was 3 nm min^−1^.

### XRD

The crystalline structure of the TmIG film with a thickness of 12 nm was characterized by using lab‐based x‐ray diffraction (Emp3, Malvern Panalytical). For the thinner TmIG film with a thickness of 6 nm, the XRD was performed on synchrotron‐based machines at the BL02U2 Beamline in Shanghai Synchrotron Radiation Facility (SSRF). The X‐rays erergy was 10 keV (λ = 0.12398 nm).

### High‐Resolution TEM

For TEM measurements, the samples were prepared using a focused ion beam instrument (Crossbeam350, Zeiss). Cross‐sectional high‐resolution TEM was performed using JEM‐F200 (JEOL) operated at 200 kV.

### Electrical Transport Measurements

AHE measurements were performed in a physical property measurement system (PPMS DynaCool, Quantum Design).

### XAS and XMCD Measurements

Typical XAS and XMCD spectra of theTmIG films were probed at the Fe‐L_3,2_ edges and Tm‐M_5,4_ edges with TEY modes at the BL07U Beamline in SSRF. A thin Pt layer (≈1.5 nm) was capped on the TmIG film as a conducting layer using DC magnetron sputtering. Opposite circularly polarized X‐rays were used to resolve the XMCD signals. The variable temperature environment (4.2 to 350 K) and the magnetic field condition (up to 9 T) were managed using the superconducting cryogenic system (Cryogenic Limited).

## Conflict of Interest

The authors declare no conflict of interest.

## Supporting information



Supporting Information

## Data Availability

The data that support the findings of this study are available from the corresponding author upon reasonable request.
