# Supplementary material for: Tailoring Unusual Ferrimagnetism in Rare‐Earth Iron Garnets via Graphene Interlayers
Source: Adv Sci (Weinh). 2025 Sep 3;12(43):e06085. doi: 10.1002/advs.202506085 (PMC12631867; doi:10.1002/advs.202506085)
Supplement: Supplementary file 1 — Supporting Information [file ADVS-12-e06085-s001.docx]

**Supporting Information**

**Tailoring unusual Ferrimagnetism in Rare-Earth Iron Garnets via Graphene Interlayers**

*Rui Yu^*^, Jiefeng Cao, Fangyuan Zhu, Xiangyu Meng, Yamei Wang, Junqin Li, Yong Wang^*^*

R. Yu, J. Cao, F. Zhu, X. Meng, Y. Wang, J. Li, Y. Wang.

Shanghai Synchrotron Radiation Facility, Shanghai Advanced Research Institute, Chinese Academy of Sciences, Shanghai 201204, P. R. China

yur@sari.ac.cn; wangyong@sari.ac.cn

**Table of Contents**

**S1.** Illustration of graphene transfer and the corresponding Raman spectra measurements.

**S2.** The estimation of the induced strain on TmIG films by graphene interlayers.

**S3.** The estimation of the effective perpendicular magnetic anisotropy fields (H_K_) in the SGGG/(0~5G)/TmIG(12 nm)/Pt samples.

**S4.** Temperature-dependent AHE measurements up to 2T.

**S5.** The calculation of the orbital and spin moments of Fe elements by using XMCD sum rules.

**S6.** Atomic multiplet claculations for magnetism contributions from Fe element *a-* and *d-sites.*

**S7.** XAS measurements of the TmIG(12 nm)/(0~5G)/SGGG samples with taken at Fe-L_3,2_ edges under grazing incidences.

**S8.** Strain-dependent *E_MA_* for the TmIG(12 nm)/(0~5G)/SGGG samples.

**S9.** The calculations resutls about strain-dependent magnetic anisotropy energy for TmIG film.

**S10.** Temperature-dependent XAS and the corresponding XMCD signals with taken at Fe-L_3,2_ edges for these samples TmIG(6 nm)/(1G), TmIG(6 nm)/(3G) and TmIG(6 nm)/(5G).

**S11.** Temperature-dependent XAS and the corresponding XMCD signals with taken at Fe-L_3,2_ edges for these samples TmIG(12 nm)/(0~5G)/SGGG.

**S12.** Temperature-dependent XAS and the corresponding XMCD signals with taken at Tm-M_5,4_ edges for these samples TmIG(6 nm)/(0~5G)/SGGG.

**S13.** Magnetic field-dependent XAS signals with taken at Fe-L_3,2_ edges for the sample TmIG(6 nm)/(2G)/SGGG.

**S14.** Temperature-dependent magnetic moments for Fe and Tm elements in TmIG(6 nm)/(2G)/SGGG sample.

**S1. Illustration of graphene transfer and the corresponding Raman spectra measurements.**

**Figure S1.** a) Schematic illustration of graphene transfer. b) Raman spectra of graphene with 1~5 layers after transferring to SGGG substrate. G and 2D peaks are identified in all the transferred graphene layers. c) Raman spectra of graphene for SGGG/1G (the wine line) and the samples SGGG/1G/TmIG(6 nm), SGGG/2G/TmIG(6 nm), SGGG/1G/TmIG(12 nm) (the blue line).

For preparing the varying layers graphene-coated SGGG substrates, the copper-based graphene has been taken and spin coating by PMMA with being 1500/min and stable 20s. After that, the PMMA is heated under 373 K for 10 mins. Then the treated graphene is immersed into the FeCl_3_ liquid for about 1 hours and the copper is all corroded away. Then graphene with PMMA is fully transferred onto (111)-oriented SGGG (0.5 mm) substrate with immersing into acetone and a single graphene has been left on the SGGG substrate. **Figure S1**a presents a schematic illustration for graphene transfer. For the bilayers graphene, we just repeat this transfer process twice, applying the same method for graphene transfer with varying layers. Meanwhile, for the 1~5 layers of graphene coated SGGG substrates, the Raman spectra are carried out and a visible G- and 2D-band are observed shown in **Figure S1**b which indicats a high-quality, defect-free graphene samples in our cases^[1-3]^. However, the two prominent peaks (G- and 2D-band as labeled in **Figure S1**c with pink arrow) disappear, indicating the degration of the quality of graphene layers after the depostion of TmIG films and high temperature post-annealing treatment.

**S2. The estimation of the induced strain on TmIG films by graphene interlayers.**

Based on the XRD presented in **Figure 1**c, the (444) peak positions (2θ) can be determined for TmIG(6nm)/(0~5G)/SGGG samples and thereby estimates the induced strain (*ε*) in the TmIG films. According to the Bragg’s law for the XRD *2dsinθ=nλ*, where *d* is the spacing between diffracting planes parallel to the film plane, *θ* is the incident angle of the X-ray, *n* was an integer, and *λ* is the wavelength of the X-ray, and then the strain could be described *ε=(d_SGGG_-d_TmIG_)/d_SGGG_=1-sinθ_SGGG_/ sinθ_TmIG_*, where *d_SGGG_* and *d_TmIG_*, *θ_SGGG_* and *θ_TmIG_* are the spacing and the peak positions, respectively^[4]^. From the **Figure 1**c, the peak positions for SGGG substrate and TmIG films with varying graphene interlayers are obtained and then the value of *ε* is estimated as a function of t_G_ shown in **Figure 1**d.

**S3. The estimation of the effective perpendicular magnetic anisotropy fields (H_K_) in the SGGG/(0~5G)/TmIG(12 nm)/Pt samples.**

**Figure S3** a) Schematic illustration of Hall bar and measurement configuration for extracting the perpendicular anisotropy filed H_K_. b) Anomalous Hall resistance (R_AHE_) as a function of external in-plane magnetic field H_X_ for the sample SGGG/2G/TmIG(12 nm)/Pt. c) The corresponding Normalized R_AHE_ as a function of H_X_. d) The ratios of H_K_ (1~5G) to H_K_ (0G) and R_AHE_ (1~5G) to R_AHE_ (0G) as a function of the layers of graphene interlayers (t_G_) for Pt/TmIG(12 nm)/(0~5G)/SGGG samples.

To extract the effective perpendicular magnetic anisotropy fields (H­_k_), we perform the hard-axis anomalous Hall resistance measurements ($R_{AHE}\propto m_{z}$), with a magnetic field being applied along the x axis. The experimental configuration is shown in **Figure S3**a. **Figure S3**b presents a representative R_AHE_ signal for the SGGG/2G/TmIG(12 nm)/Pt sample and the corresponding normalized R_AHE_ by sweeping the in-plane field is presented in **Figure S3**c. H_k_ is defined as the point where the normalized in-plane component of the magnetization, $m_{x}=\sqrt{1-m_{z}^{2}}$, reaching 0.98 as illustrated in **Figure S3**c^[5]^. For a better comparison, the ratios of H_K_ (1~5G) to H_K_ (0G) and R_AHE_ (1~5G) to R_AHE_ (0G) as a function of t_G_ are presented in **Figure S3**d (left axis) and a peak behavior is occurred, suggesting that the graphene interlayers result in an enhancement of H_K_ (R_AHE_) at a specific t_G_.

**S4. Temperature-dependent AHE measurements up to 2T.**

**Figure S4** a) The raw data for temperature-dependent AHE measurements for Pt/TmIG(6 nm)/(2G)/SGGG. b) The corresponding data with subtracting the linear background.

Temperatur-dependent AHE measurements with the magnetic field up to 2T as shown in **Figure S4** a and the corresponding data with removing the linear background as shown in **Figure S4** b.

**S5. The calculation of the orbital and spin moments of Fe elements by using XMCD sum rules.**

**Figure S5** XAS spectra of the TmIG(12nm)/SGGG sample (top). The black and red lines show the positive helicity (+𝝈) and the negative helicity (-𝝈), respectively. The violet line represents the integral of the XAS for NI. The grey line is the fitting step function and the arrows mark the parameter r derived from the integral of the XAS signal. In the bottom panel, the XMCD spectra are obtained by((-𝝈)-(+𝝈)) (the yellow line). The royal line is the integral of the XMCD and the arrows mark the parameters p and q derived from the integral of the dichroic signal.

The XMCD sum rules are employed to determine the orbital and spin moments of Fe element. The detected XAS spectra for the +σ and -σ have been divided a simple factor to ensure that pre- and post-edges region are equal as shown in **Figure S5** (top). To remove the contribution from the transition to the continuum states for XAS intensity, the spectrum is fitted by using a double-step function as shown in dashed line in **Figure S5** (top). By following the XMCD sum rules, the orbital and spin moment operator’s expectation values are determined for Fe ions as:

<L_z_>_Fe_=(4q/3r)N_h_,

<S_eff_>_Fe_=2<S_z_>+7<T_z_>=((6p-4q)/r)N_h_.

Here, N_h_ is the number of holes in the final 3d shell and assumes to be 4.7 for Fe ions. T_z_ is the z-projection of the spin-dipole operator. r refers to the integrated value of the total XAS (after removing the double-step function) as shown in blue line in **Figure S5** (top). The parameters p and q refer to the integrated values of the XMCD at L_3_-edge region and the whole energy region as figured out in **Figure S5** (bottom). Generally, the <T_z_> is considered as a negligible value for Fe ions. Therefore, by using the formular above, we can obtain the orbital and spin moment as a function of t_G_ TmIG(6 or 12 nm)/(0~5G)/SGGG samples^[6]^.

**S6. Atomic multiplet claculations for magnetism contributions from Fe element *a-* and *d-*sites.**

**Figure S6** Experimental and simulated a) XAS and b) XMCD spectra of the Fe element based on atomic multiplet calculations for the SGGG/0G/TmIG(12nm) sample. c) Site-resolved magnetic moment contributions from Fe at *a*- and *d*-sites positions as a function of the number of graphene interlayers (t_G_) in SGGG/(0~5G)/TmIG(12nm) samples.

Contributions to the magnetism of Fe elements originating from both octahedral (*a*-sites) and tetrahedral (*d*-sites) coordination environments were evaluated using the *Crispy* software-a modern graphical interface designed to compute core-level spectra via semi-empirical multiplet approaches implemented in *Quanty*^[7]^. This software was developed by Marius Retegan at the European Synchrotron Radiation Facility.

Following the references in previous studies, the experimental XAS/XMCD data were fitted using the expression:

XAS_exp_=c*(a* XAS_Fe_^3+^_(Oh)_+b* XAS_Fe_^3+^_(Td)_),

XCMD_exp_=c*(a* XCMD_Fe_^3+^_(Oh)_+b* XCMD_Fe_^3+^_(Td)_),

where XAS_exp_/XCMD_exp_ is the experimental XAS/XMCD spectrum, and XAS_Fe_^3+^_(Oh)_/XCMD_Fe_^3+^_(Oh)_, XAS_Fe_^3+^_(Td)_ /XCMD_Fe_^3+^_(Td)_ are the simulated XASXMCD spectrum, parameters a and b correspond to the relative contribution of each site, and c is an overall scaling factor.

For the simulation of XAS_Fe_^3+^_(Oh)_/XCMD_Fe_^3+^_(Oh)_ spectrum, a Slater parameter reduction of 0.8, 0.85, 0.825 was used, with a 10Dq of 1.6 eV. A Lorentzian gamma of 0.36 eV was used, with Gaussian broadening of 0.1 eV. Similarly, for the simulation of XAS_Fe_^3+^_(Td)_ /XCMD_Fe_^3+^_(Td)_ spectrum, a Slater parameter reduction of 0.45, 0.95, 0.825 was used, with a 10Dq of -0.9 eV. A Lorentzian gamma of 0.36 eV was used, with Gaussian broadening of 0.1 eV. The fitted curves are presented in **Fig S6**a and b for the sample SGGG/TmIG(12nm). The extracted spectral weight ratio of O_h_ to T_d_ is 0.536, which closely matches the expected site ratio in stoichiometric TmIG. The magnetic moment of Fe at the *a*-site and *d*-site positions as a function of t_G_ are shown in **Fig S6**c for SGGG/(0~5G)/TmIG(12nm) samples. Although the experimental data can be reasonably well fitted using the simulated XMCD spectra, strain effects are not accounted for in the simulations performed via *Crispy*. Therefore, a more rigorous model based on density functional theory should be incorporated in future studies to achieve improved fitting accuracy.

**S7. XAS/XMCD measurements of the TmIG(12nm)/(0~5G)/SGGG samples with taken at Fe-L_3,2_ edges under grazing incidences.**

**Figure S7** a) Schematic illustrations of XAS measurements of TmIG(12 nm)/SGGG with inserting graphene layers under grazing incidences (GI). b) Experimental XAS (top) and XMCD spectra (bottom) of the TmIG(12 nm)/(0~5G)/SGGG samples are taken at the Fe L_3,2_ edges in GI, respectively. c) t_G_-dependent orbital moments (top) and the ratios of m_L_ (1~5G) to m_L_ (0G) (bottom) for TmIG(12 nm)/(1~5G)/SGGG samples at GI geometry, respectively. d) t_G_-dependent spin moments (top) and the ratios of m_S_(1~5G) to m_S_ (0G) (bottom) for TmIG(12 nm)/(1~5G)/SGGG samples at GI geometry, respectively. e) t_G_-dependent total moments (top) and the ratios of m_tot_(0~5G) to m_tot_(0G) (bottom) for TmIG(12 nm)/(1~5G)/SGGG samples at GI geometry, respectively. All measurements are performed at RT.

In **Figure S7**a, it presents the schematic illustration of the experimental setup for the XAS measurements with total electron yield (TEY) mode at grazing incidences (GI). The typical XAS/XMCD spectra are shown in **Figure S7**b for TmIG(12 nm)/(0~5G)/SGGG samples. By applying the XMCD sum rules analysis, as detailed in S4, although the orbital moments exhibit a similarly anisotropic effect (top panel) in **Figure S7**c as a function of t_G_, only a relatively small variation in their value is obtained compared to the NI geometry. In contrast, the spin moments present a weak trend with respect to t_G_. These results further confirm that the anisotropic orbital moments lead to the highly modulated magnetic anisotropy in TmIG films with graphene interlayers, as anticipated by theoretical predictions^[8]^.

**S8. Strain-dependent *E_MA_* for the TmIG(12 nm)/(0~5G)/SGGG samples.**

**Figure S8** Strain-dependent *E_MA_* for the TmIG(12 nm)/(0~5G)/SGGG samples.

**S9. The calculations resutls about strain-dependent magnetic anisotropy energy for TmIG film.**

**Figure. S9**. a) Schematic illustrations of the TmIG crystal structure under compressive or tensile strain. b) Strain-dependent MAE.

To clarify the influence from the strain on the magnetic anisotropy of TmIG films, the magnetic anisotropy energy (MAE) is calculated by using the Vienna Ab initio Simulation Package (VASP). The MAE is defined as the total energy difference between magnetization along the out-of-plane [001] direction and the in-plane [100] direction, i.e., MAE=E_[001]_-E_[100]_. Therefore, a positive MAE indicates that the [100] direction (ip-plane) is the easy magnetization axis, which a negative MAE implies that the [001] direction (out-of-plane) is the easy magnetization axis, indicating the system exhibits PMA properties. The corresponding results are presented in **Fig. S9.**

**S10. Temperature-dependent XAS and the corresponding XMCD signals with taken at Fe-L_3,2_ edges for these samples TmIG(6 nm)/(1G), TmIG(6 nm)/(3G) and TmIG(6 nm)/(5G).**

**Figure S10** a), b) and c) Experimental soft XAS (top panel) and the corresponding XMCD spectra (down panel) of Fe elements for these films TmIG(6 nm)/1G, TmIG(6 nm)/(3G) and TmIG(6 nm)/(5G), respectively. All signals are taken at NI geometry.

In **Figure S10**a, b and c, temperature-dependent XAS and XMCD spectra are presented for TmIG(6 nm)/(1G, 3G and 5G)/SGGG samples. A similar trend is observed as described in the maintext. As the temperature decreases, the XAS spectra change its position at both L_3_ and L_2_ peaks. And the flipping of the orientation occurs in the XMCD spectra at a specific temperature. These results further indicate the emergence of compensated magnetic features in TmIG thin films with varying graphene layers.

**S11. Temperature-dependent XAS and the corresponding XMCD signals with taken at Fe-L_3,2_ edges for these samples TmIG(12 nm)/(0~5G)/SGGG.**

**Figure S11** a), b), c), d), e) and f) Temperature-dependent experimental soft XAS (top panel) and the corresponding XMCD (down panel) of Fe element for these films TmIG(12 nm)/ (0G~5G)/SGGG, respectively. All signals are taken at NI geometry.

In **Figure S11**a-f, temperature-dependent XAS and XMCD spectra are presented for TmIG(12 nm)/(0~5G)SGGG samples. For the samples TmIG(12 nm)/(0~3G)SGGG, the lineshapes of the XAS spectra and the orientation of the XMCD spectra remain unchanged over a wide temperature range as shown in **Figre S11**a-d. However, with increasing t_G_, a similar behavior is observed like in TmIG(6 nm)/(0~5G)SGGG samples as presented in **Figure 4**a-c and **Figure S10**a-c. The lineshapes of the XAS spectra change its position for +σ and -σ at both L_3_ and L_2_ peaks. And the orientation of the XMCD spectra is flipped at a specific temperature. The experimental results indicate that the emergence of compensate magnetic properties is significantly influenced by the layers of graphene interlayers in a thicker TmIG films.

**S12. Temperature-dependent XAS and the corresponding XMCD signals with taken at Tm-M_5,4_ edges for the sample TmIG(6 nm)/(2G)/SGGG.**

**Figure S12** a) Experimental soft XAS of Tm element for the sample TmIG(6 nm)/2G/SGGG. The data are collected by total electron yield (TEY) mode at temperature (T=20K and 200K). Insets present the zoomed-in feature at M_5_ edge. b) Temperature-dependent XMCD signals for the sample TmIG(6 nm)/2G/SGGG. Data are offset for clarity. All signals are taken at NI geometry. And the magnetic field is perpendicular to the sample surface with a value of 1 T.

TmIG films, Tm^3+^ ions occupying *c*-sites are ferromagnetically coupled to Fe^3+^ ions on *a*-sites and antiferromagnetically coupled to Fe^3+^ ions on *d*-sites. Thus, for Tm elements, temperature-dependent XAS and XMCD spectra should exhibit a resembled behavior like Fe elements in TmIG(6 nm)/(0~5G)/SGGG. In **Figure S12**a and b, it presents the XAS and XMCD spectra results for TmIG(6 nm)/(2G)/SGGG as a typical representative. The XAS spectra for +σ is located at shoulder at T=200 K and inversely the XAS spectra for -σ is located at shoulder at T=20 K at M_5_ edges. Accordingly, the corresponding XMCD spectra flip its orientation at a specific temperature. These results further confirm the emerged compensated magnetic properties in thin TmIG films.

**S13. Magnetic field-dependent XAS signals with taken at Fe-L_3,2_ edges for the sample TmIG(6 nm)/(2G)/SGGG.**

**Figure S13** a) Schematic illustrations of XAS measurements of TmIG/SGGG with inserting graphene layers. b) Experimental XAS signals of Fe element for the sample TmIG(6 nm)/2G/SGGG. The data are collected via TEY mode in normal incidences (NI) and the positive helicity (+𝝈) is chosen for the detection. The external magnetic field (H_ext_) is set at 0.8 T with being parallel of the x-ray as shown in a). The blue and the green lines are taken at T=300K and 100K, respectively. Data are offset for clarity. c) H_ext_-dependent XAS signals are taken at T=175K with H_ext_=1T, 2T, 3T, 4T, 5T, 6T. d) The corresponding amplified region of Figure S9 c) with the energy range from 707 eV to 712 eV. All signals are taken at NI geometry.

For the fully compensated REIG materials, it behaves like a static antiferromagnet at its T_M_ point and is immune to the external field^[9]^. To verify this point, we measure the XAS spectra under varying magnetic field. **In Figure S13**a, it presents the schematic of the experimental setup for the XAS measurements with TEY mode at NI geometry. And the magnetic field is perpendicular to the sample plane. As detailed in the maintext and Note 7, the XAS spectra exhibit a distinct lineshape for +σ polarization at L_3_ peak below and above its T_M_ points as shown in **Figure S13**b, resulting in a flipping of the XMCD orientation. In contrast, for the sample SGGG/2G/TmIG(6 nm), around its T_M_=175 K, the lineshape the XAS spectra for +σ polarization remain unchanged with applying the magnetic field ranging from 1T to 6T as described in **Figure S13**c and the **Figure S13**d is the amplified for the dash box. It indicates the orientation of Fe moments is stable in the same direction regardless of the external field.

**S14. Temperature-dependent magnetic moments for Fe and Tm elements in TmIG(6 nm)/2G/SGGG sample.**

**Figure S14** a) Raw XAS spectra at the Tm *M_5,4_*-edges for the SGGG/2G/TmIG(6nm) sample measured at 20K. A constant offset was removed to align the pre-edge and post-edge regions. b) XAS spectra from a) after subtracting a same linear background. c) XAS spectra from b) (black and red lines), and the non-resonant background (dashed blue line), total XAS integral (after subtracting the non-resonant background). d) XMCD spectra and total XMCD integral. e), f) and g) Totally magnetic moments (m_total_) per f.u. as a function temperature for Fe (cyan), Tm (violet) and TmIG (orange) as determined from XAS/XMCD measurements and analysis.

First, the analysis procedures are shown in **Figure S14**a-d. We remove the offset for the +σ and -σ raw data by a simple factor to ensure pre- and post-edge region (without XMCD) are equal as shown in **Figure S14**a. Then the obtained data are subtracted the background (non-resonant part) by a linear approximation as shown in **Figure S14**b. Following the methodology described in previous studies^[10-11]^, the XAS spectra were processed by subtracting a linear interpolation fitted to the off-resonance regions, as illustrated by the dashed blue line in **Figure S14**c. Moreover, the spin and orbital magnetic moments of Tm ions are calculated using the following expressions^[6]^:

$\left\langle S_{z} \right\rangle_{Tm}$=$((5p-3q)/r)N_{h}$/(2+6*($\left\langle T_{z} \right\rangle_{free}$/$\left\langle S_{z} \right\rangle_{free}$), $\left\langle L_{z} \right\rangle_{Tm}$=(2*q*/*r*)$N_{h}$.

Here $\left\langle S_{z} \right\rangle_{Tm}$, $\left\langle L \right\rangle_{Tm}$ represent the expectation values of the spin and orbital angular momentum along the Z direction, respectively. The values of $\left\langle T_{z} \right\rangle_{free}$ and $\left\langle S_{z} \right\rangle_{free}$ for Tm^3+^ are taken from the Table I in the reference^[12]^, where $\left\langle T_{z} \right\rangle_{free}$ and $\left\langle S_{z} \right\rangle_{free}$ are -0.991 and -0.471, respectively. *N_h_* denotes the number of 4*f* holes, where the value of *N_h_* is taken as 6 from the literatures^[6, 10]^. $r$ is the integrated total XAS intensity after subtracting the non-resonant background. The parameters *p* and *q* correspond to the integrated XMCD intensities at the *M_5_* and *M_4_* post-edges, respectively, as shown in **Figure S14**c and d. Therefore, by using the formular above, we can obtain temperature-dependent moments for Tm element in SGGG/2G/TmIG(6nm) sample.

Moreover, as a key characteristic feature of the fully compensated REIG materials, the total magnetization is vanishing at its T_M_ point. Thus, temperature-dependent magnetic moments (m_total_) for Fe, Tm and TmIG are obtained by using the XMCD sum rules analysis as shown in **Figure S14**e, f and g, respectively. A zero-crossing point as a function of temperature is observed which further confirm the established fully compensated properties in thin TmIG films.

**References:**

[1] S. Leontsev, P. J. Shah, H. S. Kum, J. L. McChesney, F. M. Rodolakis, M. van Veenendaal, M. Velez, R. Rao, D. Haskel, J. Kim, A. N. Reed, M. R. Page, Functional properties of Yttrium Iron Garnett thin films on graphene-coated Gd3Ga5O12 for remote epitaxial transfer, *J. Magn. Magn. Mater.* **2022**, 556, 169440.

[2] J.-B. Wu, M.-L. Lin, X. Cong, H.-N. Liu, P.-H. Tan, Raman spectroscopy of graphene-based materials and its applications in related devices, *Chem. Soc. Rev.* **2018**, 47, 1822.

[3] A. C. Ferrari, D. M. Basko, Raman spectroscopy as a versatile tool for studying the properties of graphene, *Nat. Nanotechnol.* **2013**, 8, 235.

[4] J. Ding, C. Liu, Y. Zhang, U. Erugu, Z. Quan, R. Yu, E. McCollum, S. Mo, S. Yang, H. Ding, X. Xu, J. Tang, X. Yang, M. Wu, Nanometer-Thick Yttrium Iron Garnet Films with Perpendicular Anisotropy and Low Damping, *Phys. Rev. Appl.* **2020**, 14, 014017.

[5] B. Wu, M. Jin, Z. Shao, H. Fan, J. Wen, H. Li, C. Yu, B. Liu, T. Zhou, Shape anisotropy induced field-free switching and enhancement of dampinglike field in Pt/Co/PtMn heterostructures with a wedged ultrathin antiferromagnetic PtMn layer, *Phys. Rev. B.* **2023**, 108, 054417.

[6] G. J. Omar, P. Gargiani, M. Valvidares, Z. S. Lim, S. Prakash, T. S. Suraj, A. Ghosh, S. T. Lim, J. Lourembam, A. Ariando, Room Temperature Strong Orbital Moments in Perpendicularly Magnetized Magnetic Insulator, *Adv. Funct. Mater.* **2025**, 35, 2414188.

[7] M. Retegan, Crispy: v0.8.0, *https://dx.doi.org/10.5281/zenodo.1008184.* **2024**.

[8] J. StöKhr, Exploring the microscopic origin of magnetic anisotropies with X-ray magnetic circularo dichroism spectroscopy, *J. Magn. Magn. Mater.* **1999**, 200, 470.

[9] G. Sala, P. Gambardella, Ferrimagnetic Dynamics Induced by Spin‐Orbit Torques, *Adv. Mater. Interfaces.* **2022**, 9, 2201622.

[10] L. Caretta, E. Rosenberg, F. Buttner, T. Fakhrul, P. Gargiani, M. Valvidares, Z. Chen, P. Reddy, D. A. Muller, C. A. Ross, G. S. D. Beach, Interfacial Dzyaloshinskii-Moriya interaction arising from rare-earth orbital magnetism in insulating magnetic oxides, *Nat. Commun.* **2020**, 11, 1090.

[11] D. H. Suzuki, M. Valvidares, P. Gargiani, M. Huang, A. E. Kossak, G. S. Beach, Thickness and composition effects on atomic moments and magnetic compensation point in rare-earth transition-metal thin films, *Phys. Rev. B.* **2023**, 107, 134430.

[12] Y. Teramura, A. Tanaka, T. Jo, Effect of Coulomb interaction on the X-ray magnetic circular dichroism spin sum rule in rare earths, *J. Phys. Soc. Jpn.* **1996**, 65, 3056.
